# Isolation and Biological Characteristics Study of Porcine Reproductive and Respiratory Syndrome Virus GZ2022 Strain

**DOI:** 10.3390/vetsci12070651

**Published:** 2025-07-08

**Authors:** Xinmei Yang, Bin Yu, Qing Li, Hailong Ma, Zhengjun Yu, Pei Ma, Shengnan Ruan, Xuexiang Yu, Qigai He, Wentao Li

**Affiliations:** 1 National Key Laboratory of Agricultural Microbiology, College of Veterinary Medicine, Huazhong Agricultural University, Wuhan 430070, China; xinmei@webmail.hzau.edu.cn (X.Y.); yu940262935@163.com (B.Y.); littlelate@163.com (H.M.); ruan@webmail.hzau.edu.cn (S.R.); yuxuexiang@mail.hzau.edu.cn (X.Y.); 2 Key Laboratory of Preventive Veterinary Medicine in Hubei Province, The Cooperative Innovation Center for Sustainable Pig Production, Wuhan 430070, China; 3 Wuhan Golden Dragon Group, Wuhan 430015, China; liqing@whjinlong.com; 4 Hunan Provincial Engineering Research Center for Biosecurity and Disease Control in Swine Production, Changsha 410125, China; yuzhengjun_scbio@163.com (Z.Y.); mapei_scbio@163.com (P.M.)

**Keywords:** PRRSV, GZ2022, complete genome sequencing, genetic variation, phylogenetic analysis, pathogenicity

## Abstract

PRRSV continues to evolve, complicating its epidemiological landscape in China. In this study, we isolated a novel PRRSV strain, GZ2022, from a swine farm in Guizhou Province. Subsequent analyses performed on this isolate included complete genome sequencing, phylogenetic analysis, recombination assessment, and characterization of its biological properties. Phylogenetic analysis revealed that GZ2022 clusters within Lineage 1 (NADC30-like) and features a 131-amino-acid deletion in NSP2, consistent with NADC30-derived strains. Recombination analysis identified NADC30 as the major parental strain (75% genomic contribution), with a minor recombinant region (25%) derived from the highly pathogenic HuN4 strain. In vitro growth kinetics revealed peak viral titers in Marc-145 cells at 72 h post infection (hpi). Pathogenicity was evaluated in 21-day-old piglets infected with GZ2022, the highly pathogenic PRRSV strain WUH3, or negative controls. Both infected groups exhibited typical PRRS clinical signs (fever, respiratory distress) and histopathological lesions (interstitial pneumonia, pulmonary consolidation). However, GZ2022-infected piglets exhibited attenuated virulence compared to WUH3, with reduced pulmonary hemorrhage and 0% mortality compared to 80% in the WUH3 group. Seroconversion (N-protein antibodies) was observed at 14 dpi (days post inoculation) in GZ2022-infected animals, persisting throughout the 28-day trial. Viral shedding dynamics aligned with moderate pathogenicity. These findings classify GZ2022 as a moderately virulent NADC30-like recombinant strain with partial HuN4-derived genomic regions. The emergence of such strains underscores the need for sustained surveillance of PRRSV genetic diversity and systematic evaluation of the biological properties of novel variants to refine control measures and inform vaccine development.

## Simple Summary

Porcine reproductive and respiratory syndrome (PRRS) is a significant infectious disease threatening swine production. In China, emerging NADC30-like PRRSV strains exhibit concerning features including frequent recombination events and partial immune evasion capabilities. In this study, we isolated and characterized a novel recombinant NADC30-like PRRSV strain, designated GZ2022, from a swine farm in Guizhou Province. The biological and pathogenic properties of this strain were systematically evaluated. Phylogenetic and recombination analyses revealed that GZ2022 is a recombinant virus, with NADC30 serving as the major parental strain and HuN4 as the minor contributor. In vivo experiments demonstrated that GZ2022 exhibits moderate virulence, inducing typical PRRS clinical signs in piglets, but causes less severe symptoms than highly pathogenic strains. These findings provide important insights into the molecular epidemiology and pathogenicity of PRRSV in China.

## 1. Introduction

PRRS is a major infectious disease affecting the swine industry in China [1,2,3]. It is characterized by reproductive disorders in sows and respiratory problems in pigs of all ages. Following infection with PRRSV, pigs often experience immune suppression, leading to secondary infections that severely impact herd health and productivity [4,5]. Furthermore, PRRSV evolves continuously through mutation and recombination, resulting in new strains that diminish the effectiveness of existing vaccines, complicating disease control efforts [6,7]. Despite ongoing management strategies, significant challenges in controlling PRRS persist in China.

The PRRSV genome is a single-stranded, positive-sense RNA molecule approximately 15 kb in length [8,9], characterized by a high mutation rate that enables rapid adaptation to the host and evasion of immune detection. Genetic variability and low homology between different strains can result in up to 60% genetic divergence, with divergence reaching about 10% even within the same strain [2]. PRRSV strains are diverse and are primarily categorized into European (PRRSV-1) and American (PRRSV-2) types [10,11], which differ significantly in biological characteristics and pathogenicity, complicating control strategies. Moreover, PRRSV has developed several mechanisms to escape host immune surveillance, leading to immune suppression after infection and facilitating secondary or mixed infections, particularly with other viruses or bacteria, which significantly increase mortality rates [12,13].

Recent years have witnessed frequent reports in China of a novel PRRSV strain belonging to Lineage 1. This strain shares high sequence similarity with the NADC30 strain first isolated in the United States in 2008, particularly in its amino acid (aa) deletion pattern within the nonstructural protein 2 (NSP2) region, leading to its designation as an NADC30-like strain [14,15,16]. NADC30-like strains exhibit three characteristic deletions in the NSP2 coding region: a 111-aa deletion spanning aa positions 322–432; a single-aa deletion at position 483; and a 19-aa deletion spanning positions 504–522, for a total of 131 noncontiguous aa deletions [17]. These deletions serve as key molecular markers distinguishing NADC30-like strains from other PRRSV lineages. However, both NADC30-like and highly pathogenic PRRSV (HP-PRRSV) strains are prone to recombination events, resulting in variants with significantly altered biological properties and virulence [18]. Since their emergence, NADC30-like strains have rapidly recombined with domestic PRRSV isolates and accumulated mutations, gradually replacing HP-PRRSV as the predominant lineage in Chinese swine herds [17,19]. Given the limited cross-protection afforded by commercial vaccines against these recombinant variants [20], isolating and characterizing currently circulating PRRSV strains remains essential for refining control measures and guiding vaccine development in China.

To better understand the biological characteristics and pathogenicity of currently circulating PRRSV strains, this study focused on the genetic evolution of the NADC30-like strain. A recombinant strain, designated PRRSV GZ2022, was isolated and identified, and its pathogenicity was systematically evaluated in comparison with the highly pathogenic PRRSV strain WUH3. PRRSV GZ2022 and WUH3 strains were separately inoculated into weaned piglets to investigate their pathogenicity, infection patterns, and the cross-protective ability of different sera against these strains. This research aims to provide a theoretical foundation for exploring the pathogenic mechanisms, immune protection, and vaccine development for recombinant strains of NADC30 and highly pathogenic PRRSV strains.

## 2. Materials and Methods

### 2.1. Cells, Viruses, Animals, and Ethics Statement

Porcine alveolar macrophages (PAMs) and Marc-145 cell lines, cryopreserved in liquid nitrogen, were obtained from our laboratory. Viral isolation was performed using PRRSV-positive lung tissue samples collected from pigs clinically suspected of being affected by PRRS at a swine farm in Jianhe County, Guizhou Province. The PRRSV GZ2022 strain (GenBank accession no. OR369723.1) was isolated, identified, and preserved in this study. The classical PRRSV strain R98 (accession number: DQ355796.1) and the highly pathogenic PRRSV strain WUH3 (accession number: HM853673.2) were maintained in our repository. Reference strains for phylogenetic analysis were retrieved from the NCBI GenBank database and peer-reviewed publications (full metadata are provided in Supplementary Table S1). Fifteen, 21-day-old, PRRSV antigen-negative healthy male piglets were procured from Hubei Jinlin Original Breeding Livestock Co., Ltd., Wuhan, China. All animal experiments were conducted under Animal Experimental License SYXK (E) 2020-0084 and Ethics Approval No. HZAUSW-2024-0038, in compliance with institutional guidelines for animal welfare and biosafety protocols.

### 2.2. Isolation and Identification of PRRSV Field Strains

Lung tissues from PRRS-suspected pigs were homogenized in DMEM, clarified by centrifugation (12,000× *g*, 3 min), and filtered (0.22 μm) for RNA extraction and cDNA synthesis. *ORF5* and *ORF7* genes were amplified via PCR (primers listed in Supplementary Table S2) and sequenced. PAMs were inoculated with a PCR-positive supernatant along with controls (WUH3 strain, DMEM). After a 2 h adsorption period, cells were maintained at 37 °C in 5% CO_2_ and monitored for cytopathic effects (CPEs) at 24 hpi. Virus isolation was validated through three blind passages in PAMs, followed by serial propagation in Marc-145 cells, with cryopreservation at −80 °C after CPE stabilization.

### 2.3. Viral Plaque Purification and Growth Curve Detection

Plaque assays were performed as described previously [21]. Cells were fixed with 0.1% crystal violet in 20% ethanol for 4 h at room temperature (RT), and plaque morphology was imaged under standardized conditions. All experiments were performed in triplicate. PFU/mL and MOI were calculated based on PFU and cell counts.

For viral growth curve analysis, Marc-145 cells cultured in 6-well plates were infected with PRRSV isolates at a multiplicity of infection (MOI) of 0.1. Following infection, cell supernatants were collected at 12, 24, 36, 48, 60, 72, 84, 96, 108, and 120 hpi. All experiments were performed in triplicate. Viral titers were quantified using 50% tissue culture infectious dose (TCID_50_) assays, with calculations performed according to the Reed–Muench method [22].

### 2.4. Indirect Immunofluorescence Assay (IFA)

Indirect immunofluorescence assays were performed as described by Ma et al. [23]. The cell culture supernatant exhibiting CPE was inoculated onto Marc-145 cells. At 48 hpi, cells were incubated with a murine anti-PRRSV N monoclonal antibody (this antibody was generated by the research group of Professor Zili Li at Huazhong Agricultural University), followed by an Alexa Fluor 488-conjugated goat anti-mouse IgG secondary antibody. Nuclei were counterstained with DAPI, and fluorescence signals were captured using a Nikon Eclipse Ti2 microscope under standardized exposure parameters.

### 2.5. Phylogenetic and Evolutionary Analysis

Overlapping primers (Supplementary Table S2) for full-genome amplification of strain GZ2022 were designed based on the NADC30-like PRRSV reference genome (GenBank: JN654459). Total RNA was reverse-transcribed and amplified into full-length PRRSV cDNA. Primer synthesis and Sanger sequencing were performed by Beijing Tsingke Biotechnology Co., Ltd. (Beijing, China). Genome assembly and annotation were performed using SeqMan Pro v17.0 and EditSeq v12.1.

The GZ2022 genome was phylogenetically analyzed against reference PRRSV strains (Supplementary Table S1) using (1) maximum-likelihood tree construction, (2) *NSP2* deletion profiling, and (3) comparative genomic/GP5 amino acid analyses. Evolutionary distances were derived from pairwise genome comparisons (1000 bootstrap replicates). To characterize recombination events in strain GZ2022, full-genome sequences were analyzed using SimPlot v3.5.1 and RDP5 Beta 4.101. SimPlot sliding window analysis (2000 bp window, 200 bp step) identified recombination signals between GZ2022 and potential parental strains. RDP5 was used to predict recombination sites within the full genomic sequence of the GZ2022 strain.

### 2.6. Animal Experiments

#### 2.6.1. Virus Inoculation in Piglets and Clinical Monitoring

A total of 15 piglets were randomly assigned to three groups (*n* = 5 per group). Piglets in Group 1 were simultaneously inoculated with 2 mL of the PRRSV GZ2022 strain (10^6^ TCID_50_/mL) via the intranasal route and 2 mL via intramuscular injection. Piglets in Group 2 received an identical inoculation regimen with the PRRSV WUH3 strain (10^6^ TCID_50_/mL). Group 3 served as the negative control and received 2 mL of Marc-145 cell supernatant via both the intranasal and intramuscular routes. Rectal temperatures were recorded daily for 28 dpi; body weights and clinical signs (scored daily using a standardized system: see Supplementary Table S3 for detailed criteria) were assessed at 1, 3, 5, 7, 9, 11, 13, 15, 17, and 19 dpi [24].

#### 2.6.2. Viral Load Quantification and Humoral Immune Response Assessment

Serum samples collected at 0, 1, 3, 7, 14, 21, and 28 dpi were analyzed for viral RNA via qRT-PCR (primers listed in Supplementary Table S2). Piglets that succumbed during the trial underwent immediate necropsy, while survivors were euthanized at 28 dpi. Tissues (lungs, spleen, kidneys, tonsils, and inguinal lymph nodes) were harvested for viral load quantification by qRT-PCR [25]. Serum samples (0–28 dpi) were analyzed using a PRRSV-specific ELISA kit (BioChek, Reeuwijk, The Netherlands) following the method of Lu et al. [26], with antibody levels quantified via standard curves (seropositivity cutoff: 0.4). Virus neutralization assays [27] tested GZ2022-infected piglet sera (14–28 dpi) against R98, WUH3 (HP-PRRSV), and NADC30-like strains, with neutralizing titers calculated by the Reed–Muench method.

#### 2.6.3. Necropsy and Gross Pathological Examination

Piglets that died during the experimental period underwent immediate necropsy, with major gross pathological lesions systematically documented. At the study endpoint (28 dpi), all surviving piglets were euthanized and subjected to necropsy to evaluate the pathological features of major organs, such as the lungs, in the GZ2022 infection group, WUH3 infection group, and control group.

### 2.7. Statistical Analyses

Statistical analyses were performed using Student’s *t*-test for independent samples in GraphPad Prism 10 (GraphPad Software Inc., San Diego, CA, USA). Data are expressed as mean ± standard deviation (mean ± SD). Statistical significance was defined as follows: * *p* < 0.05 (significant), ** *p* < 0.01, *** *p* < 0.001, and **** *p* < 0.0001 (highly significant); differences with *p* > 0.05 were considered nonsignificant.

## 3. Results

### 3.1. Virus Isolation and Identification

**Figure 1:**
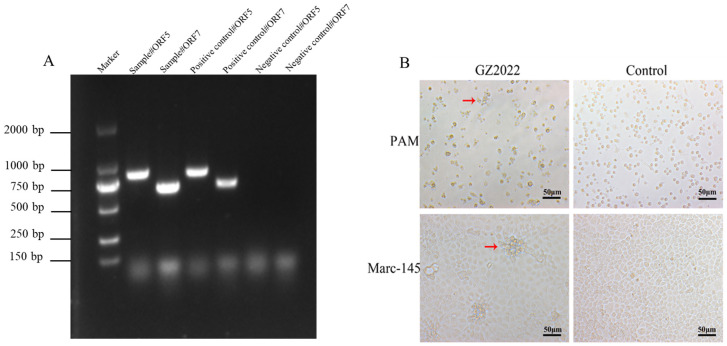
Isolation and identification of PRRSV GZ2022. (**A**) PCR identification of PRRSV GZ2022. PCR detection of PRRSV-ORF7 and PRRSV-ORF5 genes was performed on the cDNA of the samples. (**B**) CPE identification of the GZ2022 strain in PAM and Marc-145 cells. The GZ2022 strain was inoculated into PAM and Marc-145 cells, with a blank control group included. The red arrow indicates the site of cell lesion. Cytopathic effects were observed under an optical microscope at 3 to 4 dpi (CPE; 40 × magnification).

Specific amplification of the *ORF5* (957 bp) and *ORF7* (671 bp) genes via PCR and agarose gel electrophoresis confirmed PRRSV infection, with banding patterns consistent with positive controls (Figure 1A). Distinct CPEs were observed in PRRSV-inoculated cells. PAM cells exhibited pronounced shrinkage and membrane rupture, while Marc-145 cells developed characteristic grape-like clusters, a hallmark of PRRSV infection. In contrast, negative control cells retained intact morphology without pathological alterations (Figure 1B). These results demonstrate that the GZ2022 strain retains cytopathogenic capability in both PAM and Marc-145 cells, confirming its infectivity and replication efficiency in vitro.

### 3.2. Plaque Purification and Growth Curve Assay of GZ2022

**Figure 2:**
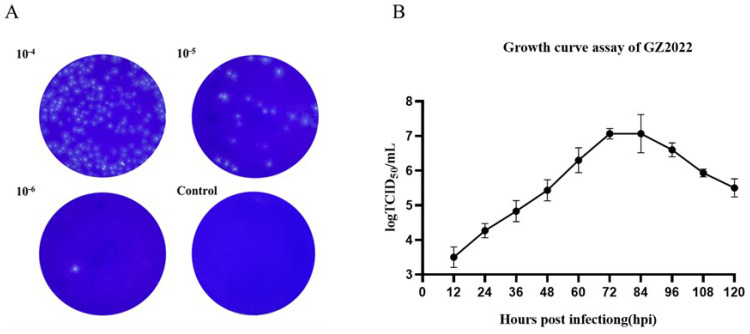
(**A**) Plaque assays of GZ2022 strain. Plaque assays were conducted using three experimental groups at dilutions of 10^−4^, 10^−5^, and 10^−6^, alongside a control group (Scale bar = 200 μm). (**B**) One step growth curve of PRRSV GZ2022 strain. The experiment was repeated three times at each time point.

Serial 10-fold dilutions (10^−2^–10^−6^) of GZ2022 were subjected to plaque assays on Marc-145 monolayers. After three rounds of plaque purification, uniform plaque morphology was observed across generations. At the 10^−6^ dilution, a single plaque was isolated, while no plaques formed in negative controls. Crystal violet staining revealed distinct plaque boundaries (Figure 2A).

The 50% tissue culture infectious dose (TCID_50_) of GZ2022 was determined to be 10^−6^/mL via the Reed–Muench method. A one-step growth curve revealed peak viral titers (10^7.1^ TCID_50_/mL) at 72 hpi in Marc-145 cells, followed by a gradual decline (Figure 2B).

### 3.3. IFA Results of GZ2022

**Figure 3:**
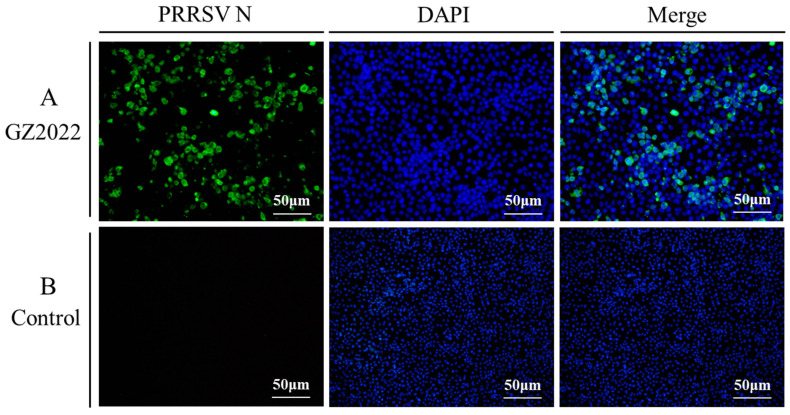
Immunofluorescence analysis of PRRSV infection in Marc-145 cells at 48 hpi. (**A**) Marc-145 cells infected with the PRRSV GZ2022 strain exhibited positive immunofluorescence staining (green) accompanied by CPEs. (**B**) No specific immunofluorescence signal was detected in mock-inoculated cells (CPE; 40 × magnification).

Immunofluorescence analysis using a monoclonal antibody targeting the PRRSV N protein revealed intense green fluorescence in Marc-145 cells infected with the GZ2022 strain (Figure 3), confirming active viral replication. DAPI counterstaining confirmed the integrity of the nuclei, with fluorescence predominantly localized to the perinuclear regions.

### 3.4. Genomic Characterization and Recombination Analysis of the GZ2022 Strain

**Figure 4:**
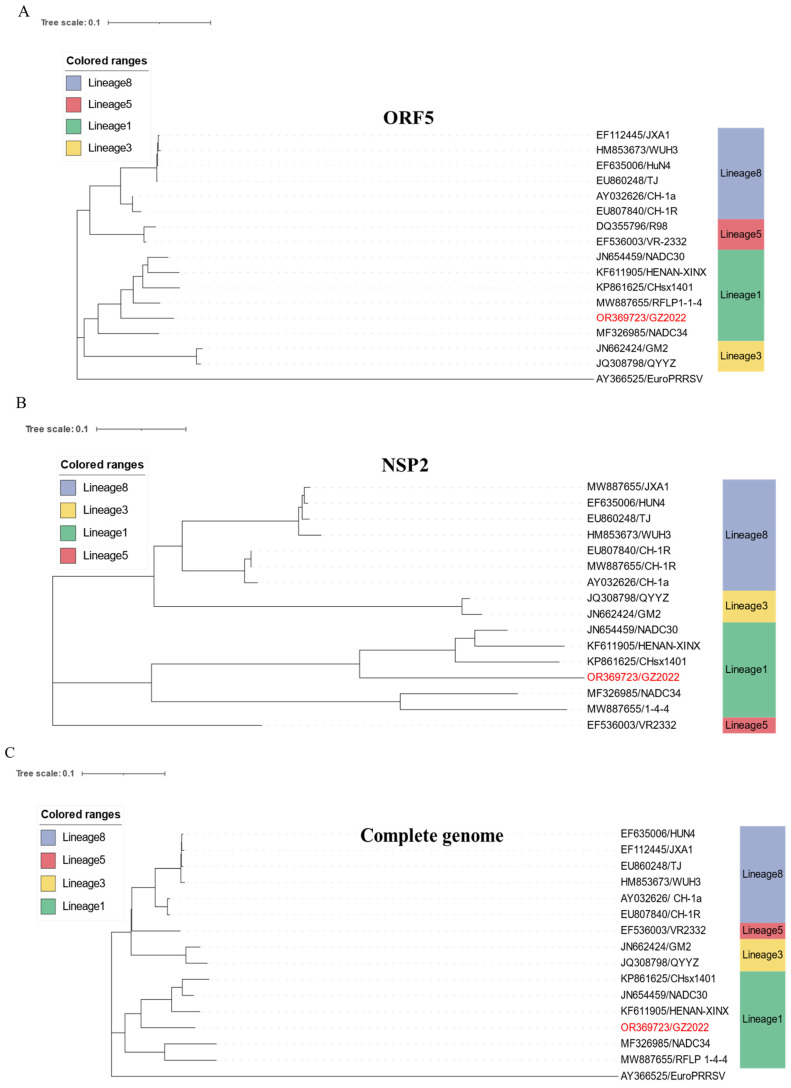

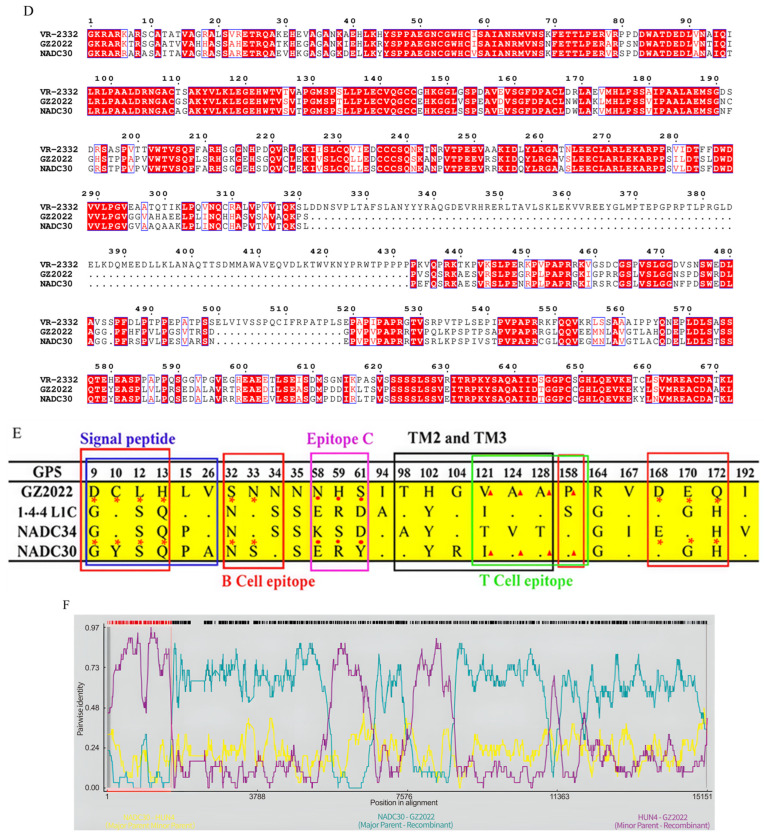

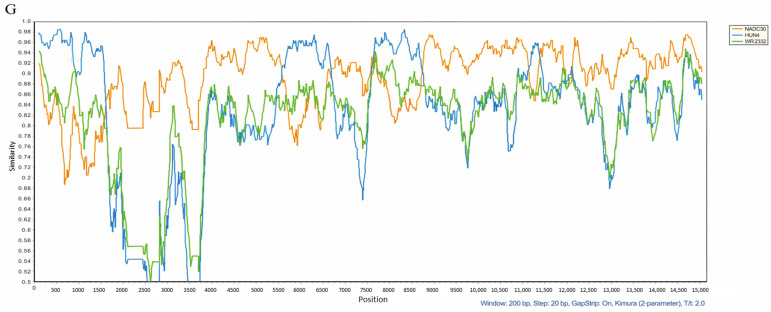
(**A**) ORF5 phylogenetic tree. (**B**) NSP2 phylogenetic tree. (**C**) Full-length genome phylogenetic tree. (**D**) NSP2 region deletion pattern analysis. Multiple sequence alignment of NSP2 sequences was conducted to identify strain-specific deletions. (**E**) GP5 amino acid analysis. The asterisk (*) denotes B-cell epitopes, the circle (○) denotes T-cell epitopes, and the triangle (Δ) denotes linear antigenic epitopes. (**F**) Recombination breakpoints identified using the RDP5 algorithm. (**G**) SimPlot analysis showing genome-wide recombination signals.

Whole-genome sequencing of the GZ2022 strain generated a 14,638 bp sequence, which was deposited in the NCBI GenBank under accession number OR369723.1. Phylogenetic analysis of the *ORF5* (Figure 4A), *NSP2* (Figure 4B), and complete genome (Figure 4C) confirmed its classification within Lineage 1 (NADC30-like strains). We performed sequence alignment of NSP2 amino acid sequences from the GZ2022 isolate and reference strains, revealing three discontinuous deletions in GZ2022 totaling 131 amino acids relative to classical VR-2332: a 111-aa deletion (positions 322–432), a 1-aa deletion (position 483), and a 19-aa deletion (positions 504–522), with this deletion topology being identical to the NADC30 reference strain as shown in Figure 4D. GP5 amino acid mutation analysis identified critical epitope mutations through comparison of GP5 protein sequences between GZ2022 and NADC30 strains (Figure 4E). Functional mutations occurred in predicted B-cell epitope regions (9–13 aa, 32–34 aa, 158 aa, 168–172 aa), with six consecutive mutations in the 9–26 aa region. Three consecutive mutations were present in both 32–34 aa and 168–172 aa epitopes. Key T-cell epitope mutations clustered in the 121–158 aa region, while the linear antigenic epitope at 58–61 aa contained three consecutive mutations. Nucleotide and amino acid homology analysis of all ORFs revealed that GZ2022 shared the highest sequence identity with NADC30 (Supplementary Table S4). Recombination analysis using RDP5 (Figure 4F) and SimPlot (Figure 4G) conclusively identified GZ2022 as an NADC30-like recombinant strain, with NADC30 (Lineage 1) as the major parental strain and the highly pathogenic HuN4 (Lineage 8) as the minor parental strain.

### 3.5. Divergent Virulence, Clinical Dynamics, and Growth Impacts of GZ2022

**Figure 5:**
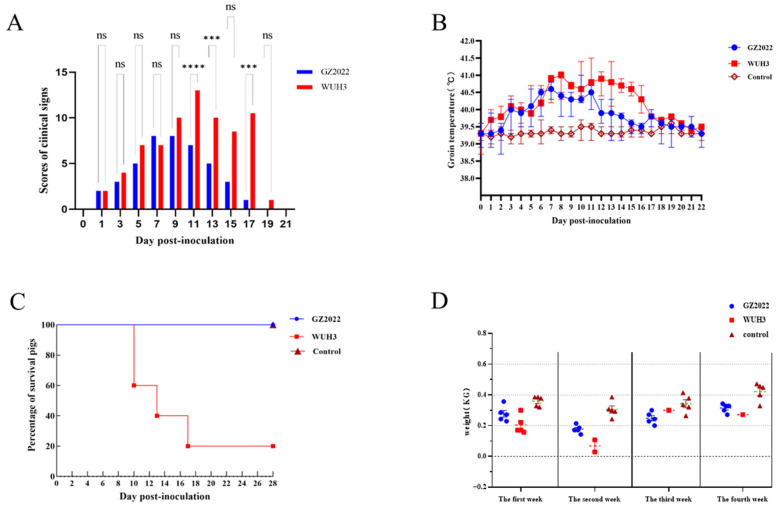
(**A**) Clinical signs score. (**B**) Temperature changes of piglets. Note: At 17–22 dpi, only one piglet survived in the WUH3 group, and thus no variation could be calculated (*n* = 1). (**C**) Survival rate of PRRSV-infected piglets. (**D**) Weekly mean weight gain of PRRSV-infected piglets. Values in are shown as mean ± SD. *** *p* < 0.001; **** *p* < 0.0001; ns: no significance.

The progression of clinical signs in PRRSV-infected piglets is summarized in Figure 5A. By 3 dpi, both infected groups exhibited early symptoms, including fever (>40.0 °C) and huddling behavior. The GZ2022-infected group reached peak clinical severity at 7 dpi, characterized by sustained hyperthermia (>40.5 °C), lethargy, anorexia, and respiratory manifestations (cough, nasal discharge, conjunctivitis), followed by complete resolution of clinical signs occurring by 17 dpi, manifested as absence of fever and restoration of normal appetite, indicative of a self-limiting disease course. In contrast, the WUH3-infected group displayed more severe pathogenic phenotypes, with exacerbated respiratory distress (abdominal breathing), hypersecretion, and neurological signs (tremors) emerging by 9 dpi. Cumulative mortality in the WUH3 group reached four cases (1, 2, and 1 death at 10, 13, and 17 dpi, respectively). The single surviving piglet in this group recovered from the observed PRRS clinical signs (including respiratory distress and fever) by 17 dpi. Control piglets remained clinically normal throughout the observation period. These findings demonstrate significant heterogeneity in virulence and disease progression between PRRSV strains.

Rectal temperatures were recorded daily from 0 to 28 dpi (Figure 5B). In the infected groups, body temperatures rose significantly starting at 1 dpi. The GZ2022-infected group reached a peak temperature of 40.6 °C at 7 dpi, followed by a gradual decline to baseline by 17 dpi. The WUH3-infected group exhibited a higher peak of 41.0 °C at 8 dpi, returning to normal levels by 20 dpi. No mortality occurred in the GZ2022 group, while the WUH3 group experienced cumulative mortality (2, 1, and 1 piglet at 10, 13, and 16 dpi, respectively; the survival curve is shown in Figure 5C). Control group temperatures remained within physiological ranges throughout the trial.

Body weight was monitored weekly until 28 dpi (Figure 5D). During the first week, daily weight gain (DWG) in the GZ2022- and WUH3-infected groups was significantly lower than in the control group, with values of 0.27 kg and 0.20 kg, respectively, compared to 0.36 kg in the controls. By week 2, DWG further decreased in the infected groups, with GZ2022 at 0.17 kg and WUH3 at 0.20 kg, compared to 0.30 kg in the control group. From week 3 onward, DWG gradually recovered in the infected groups, with GZ2022 at 0.30 kg and WUH3 at 0.24 kg, while the control group maintained a DWG of 0.34 kg. By week 4, DWG in the infected groups approached near-normal levels, with GZ2022 at 0.31 kg and WUH3 at 0.27 kg, compared to 0.42 kg in the control group.

### 3.6. Divergent Viral Shedding, Tissue Tropism, and Antibody Profiles of PRRSV Strains

**Figure 6:**
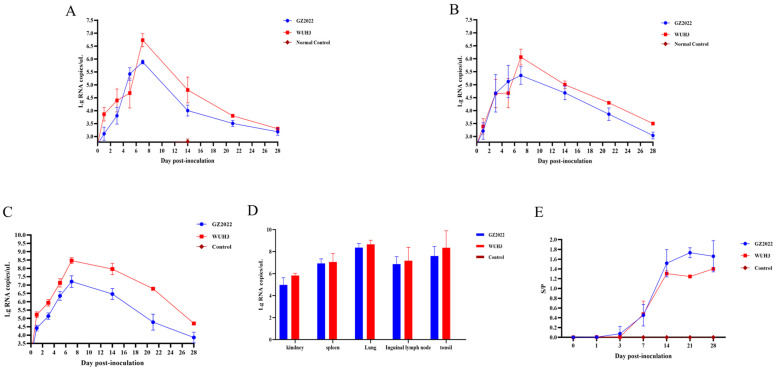
(**A**) Detection of virus shedding via nasal swabs. (**B**) Detection of virus shedding via rectal swabs. (**C**) Viral load in serum. (**D**) Viral load in tissues. Note: Viral loads in the WUH3-infected group represent individual measurements from both deceased and surviving piglets. (**E**) PRRSV antibody levels in serum. Values in are shown as mean ± SD.

Viral RNA shedding in nasal/oral and rectal swabs from infected piglets was quantitatively monitored via qPCR at multiple time points (0, 1, 3, 5, 7, 14, 21, and 28 dpi). Low levels of viral RNA were detected in the infected groups as early as 1 dpi. Nasal/oral shedding peaked at 7 dpi (Ct value = 22.1 ± 0.8, Figure 6A), while rectal shedding reached its maximum at 8 dpi (Ct value = 24.5 ± 1.2, Figure 6B). Both routes exhibited a gradual decline in viral load thereafter. Control groups remained negative throughout the experiment (Ct values > 38).

Serum viral RNA loads (Figure 6C) peaked at 7 dpi in both infected groups, with GZ2022-infected piglets showing consistently lower titers than the WUH3 group (*p* < 0.01). Viral clearance began after 7 dpi in both cohorts. Tissue-specific analysis (Figure 6D) revealed the highest viral burden in lung tissue (mean 10^7.5^ copies/g), followed by tonsils (10^6.9^ copies/g), spleen (10^6.3^ copies/g), inguinal lymph nodes (10^5.8^ copies/g), and kidney (10^5.2^ copies/g), indicating distinct organotropism of the virus.

Serum antibodies against the PRRSV N protein were quantified using a commercial ELISA kit at 0, 1, 3, 7, 14, 21, and 28 dpi (Figure 6E). Both GZ2022- and WUH3-infected groups seroconverted by 7 dpi, with mean sample-to-positive (S/P) ratios of 0.45 and 0.47, respectively (cutoff S/P ≥ 0.4). Antibody levels in the GZ2022 group peaked at 21 dpi (mean S/P = 1.7), while the WUH3 group reached maximal titers at 14 dpi (mean S/P = 1.3), followed by stabilization. Throughout the observation period, GZ2022-infected piglets exhibited higher antibody levels than the WUH3 group. All control samples remained seronegative (S/P < 0.2).

### 3.7. Gross Lesions in Piglet Lungs

**Figure 7:**
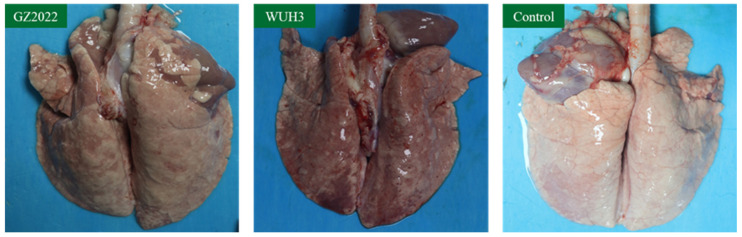
Gross lesions in piglet lungs. At 28 dpi, all surviving piglets were euthanized and necropsied to observe gross pathological lesions in lungs among the GZ2022-infected, WUH3-infected, and control groups.

Necropsy of surviving piglets at 28 dpi revealed marked pulmonary lesions in both infected groups (Figure 7). The cardiac and apical lobes of the lungs exhibited parenchymal consolidation with hemorrhagic foci; however, hemorrhagic manifestations were milder in the GZ2022 group compared with the WUH3-infected animals. The lungs of the control group maintained a pinkish hue and spongy texture, with no macroscopic abnormalities detected in other organs.

### 3.8. Pathological Analysis of Lungs and Hilar Lymph Nodes in Piglets

Serum neutralization assays were performed using sera collected from GZ2022-infected piglets at 14, 21, and 28 dpi to evaluate neutralizing antibody titers against the PRRSV classical strain (R98), the highly pathogenic strain (WUH3), and the NADC30-like strain (GZ2022) (Table 1). The results indicated that, for the NADC30-like strain (GZ2022), serum collected at 21 dpi from one piglet demonstrated a neutralizing titer of 1: 4, while at 28 dpi, sera from three piglets reached a titer of 1: 4, and one piglet’s serum reached a titer of 1: 8. For the highly pathogenic strain (WUH3) and the classical strain (R98), one piglet in each group showed a neutralizing titer of 1: 4 at 28 dpi. For the classical strain (R98), neutralizing titers remained below 1: 4 across all time points. These results indicate varying neutralizing responses to different PRRSV strains in the infected piglets.

## 4. Discussion

The emergence of novel recombinant PRRSV strains poses significant challenges to global swine health management. Since the introduction of NADC30-like strains into China in 2014, their rapid dissemination and recombination dynamics have significantly reshaped the epidemiological landscape of PRRSV [28,29,30]. This study reports the isolation and characterization of a NADC30-like recombinant strain, GZ2022, which exhibits distinctive biological properties and investigates its pathogenicity. The findings provide critical insights into the evolutionary dynamics and pathogenic mechanisms of PRRSV.

In this study, a PRRS outbreak on a pig farm in Guizhou Province was confirmed to be caused by PRRSV infection through an *ORF7* gene RT-PCR assay. Further evolutionary analysis of the ORF5 gene revealed that the strain belongs to Lineage 1 (NADC30-like clade) [31], showing the closest genetic similarity to NADC30-like strains (93.7% nucleotide identity). Notably, this strain exhibited unique adaptability to Marc-145 cells, overcoming the in vitro cultivation limitations observed in most conventional NADC30-like strains. This characteristic supports the “functional modular recombination” hypothesis proposed by Kong et al. [32], which suggests that PRRSV acquires advantageous genomic segments through cross-lineage recombination to optimize host adaptation. After three rounds of plaque purification to obtain a monoclonal virus population, whole-genome recombination analysis confirmed that GZ2022 originated from recombination between NADC30 (major parent) and HuN4 (minor parent) within the ORF1a/b region (14.3% of the genome), suggesting that structural protein exchange during recombination may enhance cellular tropism [33,34].

The *NSP2* region of GZ2022 harbors a 131-amino-acid discontinuous deletion identical to that of NADC30 [35], a feature that may attenuate its ability to activate host inflammatory responses while enhancing immune evasion [13,36]. Mutation analysis of the *ORF5* gene revealed critical substitutions in the GP5 protein at the signal peptide (G9 → D9), B-cell epitope (H172 → Q172), and neutralizing epitope (S33 → N33). These mutations likely alter antigenic epitope conformation [37] or charge distribution [17], thereby impairing the efficiency of neutralizing antibodies and limiting cross-protection [38,39]. Similar observations have underscored *ORF5* genetic variation as a key mechanism of PRRSV immune evasion [40,41].

Animal challenge experiments demonstrated that GZ2022 infection induced persistent viremia and characteristic interstitial pneumonia, yet the clinical severity was significantly milder than that induced by the highly pathogenic strain WUH3. Evaluation of pulmonary lesions remains critical for understanding PRRSV pathogenesis, as lung pathology directly reflects viral cytopathic effects and immunopathological sequelae [42]. GZ2022-infected piglets exhibited attenuated clinical signs, whereas WUH3 infection induced severe diffuse pulmonary consolidation accompanied by hemorrhage. The enhanced pathogenicity by WUH3-infected groups was attributed to accelerated replication kinetics and amplified proinflammatory cytokine induction [43], which exacerbate tissue damage despite comparable levels of viral persistence. Additionally, 7 dpi was identified as a critical window for intervention, coinciding with peak viral replication and hyperthermia, consistent with the viral kinetics model proposed by Labarque et al. [44]. ELISA detected protective N-protein antibody levels by 14 dpi; however, neutralizing antibody responses were delayed, likely due to antigenic drift resulting from GP5 epitope variation [13]. Serological analysis further revealed limited cross-neutralizing activity (maximum titer: 1: 8) against heterologous strains, reinforcing the role of antigenic drift in immune evasion [45]. Although no statistically significant differences were detected in neutralization titers between strains, likely due to the limited sample size (*n* = 5) and early-stage immune response dynamics, the observed numerical trends suggest strain-dependent variation in immunogenicity.

The Marc-145 cell adaptability of GZ2022 offers a novel resource for vaccine seed virus development; however, its antigenic diversity underscores the limitations of conventional monovalent vaccines. Designing multivalent or chimeric vaccines targeting recombination hotspots (e.g., NSP2 and ORF3) may represent a pivotal future strategy [46]. Furthermore, the establishment of a molecular surveillance network to monitor recombinant strains and track viral evolution in real time [47], coupled with reverse genetics approaches to dissect the functional contributions of specific recombinant fragments [48,49], is strongly recommended.

In conclusion, the discovery of GZ2022 not only enriches the genetic database of PRRSV recombinant strains but also provides novel insights into viral evolution and host–pathogen interactions. Future studies should prioritize elucidating the mechanisms of immune evasion and cross-lineage transmission risks associated with recombinant strains, in order to develop precision-based interventions and mitigate the global threat of PRRS to swine production.

## 5. Conclusions

This study suggests that recombination events contribute to the genetic diversification of NADC30-like PRRSV strains in China. The recombinant GZ2022 strain, classified within Lineage 1, retained key NADC30-derived genomic features (e.g., a 131-amino-acid deletion in *NSP2*) while acquiring HuN4-derived segments that enhance its adaptability to Marc-145 cells. In vivo experiments demonstrated moderate virulence, inducing typical PRRS clinical manifestations in infected piglets, albeit with attenuated severity compared to highly pathogenic strains. Although GZ2022 infection elicited neutralizing antibodies in piglets by 21 dpi, sera exhibited limited cross-neutralization against heterologous PRRSV isolates, underscoring the strain-specific nature of immune responses. These findings highlight the recombinant genomic architecture and intermediate pathogenicity of GZ2022, providing valuable insights for refining vaccine development strategies against evolving PRRSV variants.

## Figures and Tables

**Table 1:** Positive rate of the neutralization test against different strains of PRRSV.

Day	NADC30-Like Strain (GZ2022)			Highly Pathogenic Strain (WUH3)			PRRSV Classical Strain		
	1: 2	1: 4	1: 8	1: 2	1: 4	1: 8	1: 2	1: 4	1: 8
14	0	0	0	0	0	0	0	0	0
21	40% (2/5)	20% (1/5)	0	20% (1/5)	0	0	20% (1/5)	0	0
28	60% (3/5)	40% (2/5)	20% (1/5)	20% (1/5)	20% (1/5)	0	20% (1/5)	0	0

## Data Availability

The data presented in this study are available on request from the corresponding author.

## Supplementary Materials

The following supporting information can be downloaded at: https://www.mdpi.com/article/10.3390/vetsci12070651/s1, Table S1: Information of PRRSV reference strains; Table S2: Primers and probes used in this study; Table S3: Scoring criteria of clinical symptoms; Table S4: The whole genome nucleotide sequence homology analysis of GZ2022 strain.
