# Supplementary material for: Isolation and Biological Characteristics Study of Porcine Reproductive and Respiratory Syndrome Virus GZ2022 Strain
Source: Vet Sci. 2025 Jul 8;12(7):651. doi: 10.3390/vetsci12070651 (PMC12299216; doi:10.3390/vetsci12070651)
Supplement: Supplementary file 1 [file vetsci-12-00651-s001.zip › vetsci-3686802-supplementary.pdf]

Supplementary Table S1 Information of PRRSV reference strains.

Strains	Isolation Time/Year	Country	GenBank Accession Number
**VR-2332**	1990	USA	EF536003
**EuroPRRSV**	2003	USA	AY366525
**CH-1a**	1996	China	AY032626
**CH-1R**	2008	China	EU807840
**TJ**	2006	China	EU860248
**NADC30**	2008	USA	JN654459
**NADC34**	2017	USA	MF326985
**GM2**	2011	China	JN662424
**QYYZ**	2011	China	JQ308798
**CHsx1401**	2014	China	KP861625
**HENAN-XINX**	2013	China	KF611905
**RFLP 1-4-4**	2000	USA	MW887655
**JXA1**	2006	China	EF112445
**HuN4**	2006	China	EF635006
**WUH3**	2008	China	HM853673

Supplementary Table S2. Primers and probes used in this study.

Genes	Sequences (5′-3′)	Amplicons (bp)
ORF5	F-TGAAAAGGGATTCAAGGTGATATTTGG<br>R-AGAAAAATCAAGAGGTGCAAGAGC	957bp
ORF7	F-TTGCCCATTTTCAGAGTACAAACAGG<br>R- AAGCGCACGGTGTGATTAGT	671bp
**whole genome 1**	F-CATAGCTGATTGCCCGAATTGG<br>R-TATTCTTTTTAATTTCTCTACTTCTTTGGCCGT	1840
**whole genome 2**	F-CGGTCTTGTTTCCCCGGAA<br>R-CTTGAACAAACCAACAGCAAAAGC	1939
**whole genome 3**	F-TGGGGTTTTGCAGCTTTTACTTT<br>R-AAAAATGGCCTGAGGGGC	2110
**whole genome 4**	F-CACGTGCACCCCCAATG<br>R-CGGGCGAGCTTTCTCAAG	1960
**whole genome 5**	F-CTTGGGAGTGCCTCAGGG<br>R-GGGGAAACTGTAACCGTCTTTG	2170
**whole genome 6**	F-GTTGAGCATCTGAATTTGATGCTG<br>R-GGTATTTGGAGGTAACGTGATGACAT	2125
**whole genome 7**	F-CAAATGCTTCCGGAGACGG<br>R-CCATGATACGTTCAAAACCAACCA	2064
**whole genome 8**	F-ATCGATGTGAGGAGAGCGATCA<br>R-AAGCGCACGGTGTGATTAG	2296

Supplementary Table S3. Scoring criteria of clinical symptoms.

	Symptom Type	Scoring Criteria	Score
**Gross clinical score GCS**	Body temperature	T ≤ 39.9℃	0
		40.0℃ ≤ T ≤ 40.9℃	1
		41.0℃ ≤ T	2
	Appetite	Normal	0
		Anorexia	1
	Mental status	Normal	0
		Disorientation/coma	1
	Skin condition	Normal	0
		Cyanosis	1
**Respiratory clinical score RCS**	Respiratory symptoms	Normal	0
		Dyspnea during stress	1
		Dyspnea at rest	2
		Severe dyspnea with labored breathing	3
		Life-threatening respiratory distress	4
	Cough	Normal	0
		Cough	1
	Nasal discharge	Normal	0
		Nasal discharge	1
**Nervous sings score NSS**	Neurological symptoms	Normal	0
		Tremor	1
		Ataxia	2
		Limb paddling	3
		Paralysis	4

Overall Clinical Score: GSC+RCS+NSS; Death cases = Total score + 5 (max score: 20)

Supplementary Table S4. The whole genome nucleotide sequence homology analysis of GZ2022 strain.

**GZ2022**	***ORFs***	***ORF1a***	***ORF1b***	***NSP2***	***ORF2***	***ORF3***	***ORF4***	***ORF5***	***ORF6***	***ORF7***
**VR2332**	nt%	77.8	86.7	58.0	86.6	81.8	87.4	85.7	89.1	89.4
	aa%	77.7	95.1	47.8	85.6	81.2	85.5	85.1	92.6	92.9
**NADC30**	nt%	88.9	93.1	91.8	90.0	91.2	95.1	92.4	95.2	92.7
	aa%	89.0	97.5	84.9	88.3	89.8	95.5	91.0	94.9	94.7
**NADC34**	nt%	80.5	87.5	76.4	83.8	84.8	93.5	88.6	92.6	91.2
	aa%	80.8	96.0	64.8	82.1	82.7	93.9	90.0	92.6	93.8
**QYYZ**	nt%	74.2	85.5	51.9	86.4	80.1	86.1	83.7	88.2	87.1
	aa%	73.3	94.6	40.0	87.2	82.0	86.0	82.6	92.6	91.2
**GM2**	nt%	74.4	86.1	51.7	86.1	80.1	86.6	83.4	88.4	85.3
	aa%	73.6	95.3	39.8	86.8	81.6	86.6	82.1	92.6	87.6
**1-4-4**	nt%	81.5	87.4	76.0	82.6	84.3	91.3	91.5	93.5	92.7
**L1C**	aa%	81.0	95.9	65.7	80.2	80.8	90.5	92.0	94.3	94.7
**CH-1a**	nt%	78.3	88.0	56.8	87.2	81.3	87.2	86.2	87.2	88.6
	aa%	76.9	95.2	46.8	87.5	80.0	85.5	85.1	91.4	93.8
**JXA1**	nt%	78.6	87.7	57.8	86.6	81.0	87.0	85.9	87.4	87.7
	aa%	78.4	95.6	47.7	85.6	78.8	85.5	86.6	92.6	92.0
**HuN4**	nt%	78.7	87.6	57.7	86.6	81.0	87.4	86.1	87.4	87.7
	aa%	78.4	95.8	47.7	85.2	79.2	87.2	87.1	92.6	92.0
